# Concordance of disk diffusion, broth microdilution, and whole‐genome sequencing for determination of in vitro antimicrobial susceptibility of *Mannheimia haemolytica*

**DOI:** 10.1111/jvim.15883

**Published:** 2020-09-07

**Authors:** Emily R. Snyder, Bridget J. Savitske, Brent C. Credille

**Affiliations:** ^1^ Food Animal Health and Management Program, Department of Population Health, College of Veterinary Medicine University of Georgia Athens Georgia USA

**Keywords:** antimicrobial resistance, antimicrobial stewardship, bovine respiratory disease, genomics

## Abstract

**Background:**

Extensive drug resistance (XDR) is an emerging concern with *Mannheimia haemolytica*, and a variety of testing methods are available for characterizing in vitro antimicrobial susceptibility.

**Objectives:**

To compare the concordance among disk diffusion, broth microdilution, and whole genome sequencing (WGS) for susceptibility testing of *M. haemolytica* before and after mass treatment using tulathromycin.

**Animals:**

Forty‐eight *M. haemolytica* isolates collected from high‐risk beef stocker calves before and after mass treatment (metaphylaxis) using tulathromycin (Draxxin, Zoetis, Parsippany, NJ) given at the label dosage of 2.5 mg/kg body weight SC in the neck.

**Methods:**

In vitro antimicrobial susceptibility was determined for all 48 isolates using disk diffusion, broth microdilution, and WGS. Concordance was calculated between pairs of susceptibility testing methods as follows: number of isolates classified identically by the 2 testing methods for each timepoint, divided by the number of isolates tested at that timepoint. Discordance was calculated as follows: number of isolates classified differently by the 2 testing methods for each timepoint, divided by the number of isolates tested at that timepoint.

**Results:**

Concordance between testing methods ranged from 42.3% to 100%, depending on antimicrobial evaluated, timing of sample collection, and testing method used. Very major errors were identified in up to 7.7% of classifications whereas minor errors were seen in up to 50% of classifications depending on antimicrobial evaluated, timing of sample collection, and testing method used.

**Conclusions and Clinical Importance:**

Our results show that discrepancies in the results of different susceptibility testing methods occur and suggest a need for greater harmonization of susceptibility testing methods.

**Abbreviations**
AST: antimicrobial susceptibility testing
BRD: bovine respiratory disease
*Mh*: Mannheimia haemolytica
MIC: minimum inhibitory concentration
WGS: whole genome sequencing
XDR: extensively drug resistant

## 1 INTRODUCTION

Choosing the right antimicrobial can mean the difference between success and failure, health and disease, and even life and death in cattle with bovine respiratory disease (BRD). However, in the recent past, the bacterial pathogen most often associated with BRD in beef cattle, *Mannheimia haemolytica* (*Mh*), has begun to develop resistance to an increasing number of antimicrobials.^1^, ^2^ Currently, it not uncommon to see strains of this microbe resistant to ≥3 classes of antimicrobials, which has hindered implementation of effective treatment protocols.^2^, ^3^, ^4^ Veterinarians have long relied on the results of antimicrobial susceptibility testing (AST) to facilitate decision making for the design of effective treatment regimens. Disk diffusion, concentration gradient agar diffusion (E‐test), and broth macro or microdilution are methods that have commonly been used for assessing phenotypic susceptibility, with surveys showing that disk diffusion is the methodology employed by most laboratories.^5^, ^6^, ^7^ Whole genome sequencing (WGS) has been used to evaluate mechanisms and epidemiology of antimicrobial resistance at the molecular level and has potential for use in evaluating susceptibility at the genotypic level to complement the currently available phenotypic tests.^8^, ^9^, ^10^ In addition, WGS can provide information about the epidemiology of pathogen spread and provide a better understanding of genetic diversity and distribution of specific traits within a bacterial population.^11^ Although WGS has encouraging potential, it is currently not known how genotypic resistance agrees with phenotypic resistance in *Mh*. Also, it is not fully understood how the more commonly used AST methods compare for the testing of field isolates with different susceptibilities and genetic backgrounds.

In studies from the human medical literature, WGS technologies have performed very well relative to standard testing methods. One study analyzed 1379 isolates of *Staphylococcus aureus* obtained from British hospitals and found that the 3 methods of genotypic susceptibility prediction assessed agreed with culture‐based phenotypic assessment in 98.3% of cases.^12^ Another study found 99% agreement between genotypic and phenotypic susceptibility in 640 *Salmonella* spp. isolates collected from retail meat samples and human clinical cases.^13^ Numerous other examples of the high concordance among different methodologies exist and suggest that WGS technologies have utility in the assessment of antimicrobial susceptibility.^14^, ^15^, ^16^, ^17^

In the veterinary medical literature, work evaluating the utility of WGS for antimicrobial susceptibility testing of *Mh* is limited.^18^ Therefore, our goals were 2‐fold. First, we sought to assess concordance among disk diffusion, WGS, and broth microdilution for determining the in vitro antimicrobial susceptibility of *Mh* obtained from high‐risk beef stocker calves before and after mass medication using tulathromycin. Second, because studies have shown that the susceptibility of *Mh* can change markedly after mass medication, we sought to evaluate how different susceptibility testing methodologies performed in these genetically distinct populations.^3^, ^9^

## 2 MATERIALS AND METHODS

### 2.1 Isolate selection

The isolates used in this study were collected as part of a previous investigation into the prevalence of multidrug resistant *Mh* in high risk stocker cattle.^3^ Briefly, the isolates were cultured from deep nasopharyngeal swabs (NPS) collected from calves (n = 20) that were *Mh* culture‐positive at both arrival and 10 to 14 days later at revaccination, and a maximum of 3 isolates could be selected from a given time point if they all had unique antimicrobial susceptibility profiles. This approach yielded 48 isolates, 26 isolates from the calves at arrival, and 22 at revaccination. Any calves displaying signs of BRD at the time of arrival processing were excluded from the study. No calves with isolates included here were diagnosed with BRD during the course of the study.

### 2.2 Disk diffusion

The disk diffusion susceptibility data utilized in this study was obtained from susceptibility data performed as part of a previous work.^3^ All disk diffusion susceptibility testing was performed by the University of Georgia Veterinary Diagnostic Lab in Athens, Georgia. Briefly, NPS were soaked in sterile saline, streaked onto sheep blood agar plates (BAP), and incubated for 18 to 24 hours at 35°C in 5% CO_2_. Colonies with slight hemolysis and morphology consistent with *Mh* were confirmed by matrix‐assisted laser desorption ionization time‐of‐flight mass spectrometry (MALDI‐TOF MS; bioMerieux Vitek, Durham, NC). Samples from all *Mh* isolates identified were frozen in brain‐heart infusion (BHI) broth with 15% glycerol (Hardy Diagnostics, Santa Maria, California) and archived for later analysis. Selected *Mh* isolates were grown to a 0.5 McFarland standard in Mueller‐Hinton broth and streaked onto BAP. Antimicrobial impregnated disks were placed onto the plate, and zone diameters were measured at 18 to 24 hours using a Biomic V3 instrument (Biomic V3, Giles Scientific Inc., Santa Barbara, California) after incubation at 35°C in 5% CO_2_. The following antimicrobials were evaluated: ceftiofur, enrofloxacin, florfenicol, gamithromycin, tilmicosin, and tulathromycin. For each antimicrobial agent, isolates were characterized as susceptible (*S*), intermediate (*I*), or resistant (*R*) according to guidelines established by the Clinical Laboratory Standards Institute (CLSI).^19^ Diagnostic laboratory quality control was performed on a weekly basis utilizing *S. aureus* ATCC 25923 and *Escherichia coli* ATCC 25922. Interpretive criteria for disk diffusion and corresponding breakpoints are presented in Table 1. Results of AST are only reported for those antimicrobials with CLSI defined breakpoints for disk diffusion.

**TABLE 1:** CLSI interpretive criteria for disk diffusion and broth microdilution for *Mannheimia haemolytica*, with interpretation indicated as susceptible (*S*), intermediate (*I*), and resistant (*R*)

	Susceptibility breakpoints					
Antimicrobial	Disk zone interpretive criteria (mm)			Broth dilution interpretive criteria (μg/mL)		
	*S*	*I*	*R*	*S*	*I*	*R*
FLOR	≥19	15‐18	≤14	≤2	4	≥8
CEF	≥21	18‐20	≤17	≤2	4	≥8
PEN	–	–	–	≤0.25	0.5	≥1.0
OXY	–	–	–	≤2	4	≥8
ENR	≥21	17‐20	≤16	≤0.25	0.5‐1	≥2
DAN^a^	≥22	18‐21	≤17	≤0.25	0.5	≥1
TIL	≥14	11‐13	≤10	≤8	16	≥32
TUL	≥18	15‐17	≤14	≤16	32	≥64

Abbreviations: CEF, ceftiofur; CLSI, Clinical Laboratory Standards Institute; DAN, danofloxacin; ENR, enrofloxacin; FLOR, florfenicol; OXY, oxytetracycline; PEN, penicillin; TIl, tilmicosin; TUL, tulathromycin.

^a^ Interpretive criteria for intermediate and resistant ranges were not established at the time Kirby‐Bauer susceptibility was performed.

### 2.3 Broth microdilution

*Mannheimia haemolytica* test isolates were selected from archived frozen stock samples. *Staphylococcus aureus* ATCC 29213, *E. coli* ATCC 25922, and *Mh* ATCC 33396 (for tulathromycin quality control [QC] only) were used as QC isolates. All isolates were plated onto BAP for primary subculture and incubated aerobically for 18 to 24 hours at 37°C; a day 1 working culture was made from this growth and incubated in the same manner. Day 1 BAP were transported to the USDA‐ARS U.S. National Poultry Research Center in Athens, Georgia for microdilution susceptibility testing. Broth microdilution was performed using the TREK Diagnostics Sensititre System (TREK, Thermofisher Scientific, Oakwood Village, Ohio) on the Sensititre Bovine/Porcine panel with tulathromycin (BOPO6F) according to the manufacturer's directions using protocol 013‐VET‐CID9634 (TREK, Thermofisher Scientific, Oakwood Village, Ohio). Interpretive criteria for broth microdilution are presented in Table 1. Results of AST are only reported for antimicrobials with defined CLSI breakpoints for broth microdilution.

### 2.4 Whole genome sequencing

Bacterial DNA was extracted from the isolates using a commercially available kit (Ultraclean Microbial DNA Isolation Kit, Qiagen, Germantown, Maryland), and assessed for purity and concentration using the NanoDrop spectrophotometer (ThermoFisher Scientific, Waltham, Massachusetts); all samples had A260/A280 values >1.80. The extracted DNA was submitted for WGS using Illumina NextSeq (Illumina, San Diego, California). The genomic data were submitted to a consultant for cleaning and assembly. The program FastQC version 0.11.5 was used to assess initial quality, followed by Trimmomatic version 0.36 to trim the reads, with all reads having < 50 base pairs removed; FastQC was run a second time to assess trimmed read quality.^20^, ^21^ Coverage for isolates ranged from 144 to 369X, with an average coverage of 253X.

De novo assembly, using paired end reads, was performed using SPAdes version 3.11.1, and quality of the assemblies assessed using QUAST version 4.5.^22^, ^23^ Assemblies were individually queried using BLASTn against the National Center for Biotechnology Information (NCBI) database to identify *Mh* isolates with which they shared the greatest homology, and then aligned to these reference sequences using MAUVE 2.4.0 to place the scaffolds into a more biologically relevant orientation before annotation.^24^, ^25^ The 3 Genbank *Mh* strains that the assemblies aligned most closely with were D171 (CP006573.1), 89010807N (CP011098.1), and USDA‐ARS‐MARC‐185 (CP004753.2). Annotation of the ordered assemblies was performed using RASTt version 1.3.0.^26^ Resistance genes from both the Comprehensive Antibiotic Resistance Database (CARD) and from the Microbial Ecology Group Antibiotic Resistance Database (MEGARes) were queried against the selected *Mh* isolate assemblies using BLASTn.^24^, ^27^, ^28^ Additional known resistance genes not present in the aforementioned databases were downloaded from Genbank and include in the query. All resistance gene sequences identified in the isolates by BLAST were manually verified for gene length, presence of start and stop codons, and errors caused by database misidentification. The genes *gyrA* and *parC* from the known fluoroquinolone susceptible isolate *Mh* M42548 (CP005383.1) additionally were queried against the isolate assemblies to identify any point mutations known to be associated with resistance to this antimicrobial. These specific point mutations are S83F, S83Y, A84P, D87G, and D87N in *gyrA*, and S80I, S80L, and E84K in *parC*.

### 2.5 Data analysis

For broth microdilution, the concentrations required to inhibit the growth of 50% (MIC_50_) and 90% (MIC_90_) of the isolates tested were calculated for each antimicrobial agent. With each antimicrobial agent, the results of testing obtained by the 3 different methods were converted to qualitative categories. For broth microdilution and disk diffusion, qualitative categories included susceptible, intermediate, or resistant. For WGS, qualitative categories included only susceptible or resistant, based on the presence or absence of specific genes associated with resistance. Concordance was calculated between pairs of susceptibility testing methods as follows: number of isolates classified identically by the 2 testing methods, divided by the number of isolates tested. Discordance was calculated as follows: number of isolates classified differently by 2 testing methods, divided by the number of isolates tested. In the case of penicillin and tetracycline, comparisons were made only between broth microdilution and WGS because no breakpoints for these antimicrobials have been validated for disk diffusion. For gamithromycin, comparisons were made only between disk diffusion and WGS, because this antimicrobial was not routinely included on the commercially available microdilution panel at the time of study completion. Categorical discrepancies were recorded as minor (intermediate result was obtained by only 1 of the methods compared), major (isolate classified as susceptible by reference method and interpreted as resistant by the comparator), and very major (isolate classified as resistant by reference method categorized as susceptible by the comparator) errors. The broth microdilution test was considered the reference method for these determinations except when comparing WGS to disk diffusion, where disk diffusion was considered the reference method. In this scheme, very major errors would be those in which an isolate is classified as falsely susceptible relative to the reference method, and a major error would be those in which an isolate is falsely identified as resistant relative to the reference method. Generally speaking, very major errors are considered to be worse because they could lead to the use of an antimicrobial that is ineffective, resulting in increased risk of treatment failure. For a given antimicrobial, the percentage of isolates classified as susceptible, intermediate, or resistant were compared among the different testing methods using an exact test of marginal homogeneity. For all analyses, a value of *P* < .05 was considered significant. All analyses were performed using commercially available statistical software (Stata, Version 1.51, StataCorp, LP, College Station, Texas).

## 3 RESULTS

A summary of the disk diffusion susceptibilities of the 48 selected isolates is presented in Table 2. Isolates collected from calves at arrival processing were routinely susceptible to florfenicol, ceftiofur, enrofloxacin, and gamithromycin. In contrast, isolates collected from the same calves at revaccination 10 to 14 days later were consistently resistant to enrofloxacin, tilmicosin, gamithromycin, and tulathromycin, whereas they retained susceptibility to ceftiofur and susceptibility to florfenicol varied.

**TABLE 2:** Summary of disk diffusion antimicrobial susceptibility test results for 48 *Mannheimia haemolytica* isolates collected from 20 stocker calves before (Arrival, n = 26) and 10 to 14 days after (Revacc, n = 22) metaphylactic treatment with tulathromycin

No. isolates (%)				
Antimicrobial	Occasion	Susceptible	Intermediate	Resistant
FLOR	Arrival	26 (100%)	0	0
	Revacc	13 (59.1%)	1 (4.5%)	8 (36.4%)
CEF	Arrival	26 (100%)	0	0
	Revacc	22 (100%)	0	0
ENR	Arrival	26 (100%)	0	0
	Revacc	0	0	22 (100%)
TIL	Arrival	19 (73.1%)	7 (26.9%)	0
	Revacc	0	0	22 (100%)
GAM	Arrival	26 (100%)	0	0
	Revacc	0	0	22 (100%)
TUL	Arrival	21 (80.8%)	5 (19.2%)	0
	Revacc	0	0	22 (100%)

Abbreviations: CEF, ceftiofur; ENR, enrofloxacin; FLOR, florfenicol; GAM, gamithromycin; TIL, tilmicosin; TUL, tulathromycin.

Minimum inhibitory concentration distributions of the selected isolates as determined by broth microdilution are presented in Table 3. As with disk diffusion, isolates collected from calves at the time of arrival processing were routinely susceptible to most of the antimicrobials evaluated. In addition, isolates collected from calves at revaccination were routinely resistant to most antimicrobials evaluated. The major exceptions were consistent susceptibility to ceftiofur and variable susceptibility to florfenicol.

**TABLE 3:** Broth microdilution resistance breakpoint (μg/mL), MIC_50_ and MIC_90_ values, and percentage resistant of 48 *Mannheimia haemolytica* isolates collected at the arrival (n = 26) and 10 to 14 days later at revaccination (n = 22)

Antimicrobial	Timepoint	Resistant breakpoint (μg/mL)	MIC_50_ (μg/mL)	MIC_90_ (μg/mL)	% resistant
FLOR	Arrival	≥8.0	0.5	1	0
	Revacc		4	≥8	40.9%
CEF	Arrival	≥8.0	≤0.25	≤0.25	0
	Revacc		≤0.25	≤0.25	0
PEN	Arrival	≥1.0	0.5	0.5	7.7%
	Revacc		≥8	≥8	86.3%
OXY	Arrival	≥8.0	≤0.5	≤0.5	3.8%
	Revacc		≥8	≥8	100%
ENR	Arrival	≥2.0	≤0.12	≤0.12	0
	Revacc		≥2	≥2	100%
DAN	Arrival	≥1.0	≤0.12	≤0.12	0
	Revacc		≥1	≥1	100%
TIL	Arrival	≥32.0	8	16	3.8%
	Revacc		≥64	≥64	100%
TUL	Arrival	≥64.0	4	8	0
	Revacc		≥64	≥64	95.5%

Abbreviations: CEF, ceftiofur; DAN, danofloxacin; ENR, enrofloxacin; FLOR, florfenicol; OXY, oxytetracycline; PEN, penicillin; TIL, tilmicosin; TUL, tulathromycin.

The genes and mutations identified in the isolates by whole WGS BLAST queries, the antimicrobial class for which they encode resistance, and the proportion of isolates positive for each gene at the 2 sampling time points are presented in Table 4. Genes and mutations identified include the phenicol resistance gene *floR*, β‐lactamases *bla‐ROB1* and *bla‐OXA2*, tetracycline resistance genes *tetH*/*R*, macrolide resistance genes *erm42*, *msrE* and *mphE*, as well as fluoroquinolone resistance mutations *gyrA* S83F, *gyrA* D87N, and *parC* E84K.

**TABLE 4:** Resistance genes identified by BLAST query of 48 *Mannheimia haemolytica* whole genome sequences, with total number and percentage of isolates positive at arrival (n = 26) and 10 to 14 days later at revaccination (n = 22)

Antimicrobial class	Gene	Timepoint	Gene positive (%)
Phenicols	*floR*	Arrival	0
		Revaccination	8 (36.4%)
β‐Lactams	*bla*‐*OXA2*	Arrival	0
		Revaccination	22 (100%)
	*bla*‐*ROB1*	Arrival	1 (3.8%)
		Revaccination	18 (81.8%)
Tetracyclines	*tetH*/*R*	Arrival	1 (3.8%)
		Revaccination	22 (100%)
Fluoroquinolones	*gyrA* S83F *gyrA* D87N *parC* E84K	Arrival	0
		Revaccination	22 (100%)
Macrolides	*erm42* *msrE* *mphE*	Arrival	0
		Revaccination	22 (100%)

### 3.1 Disk diffusion vs broth microdilution

Concordance between disc diffusion and broth microdilution is presented in Table 5. For florfenicol, concordance between the methods for arrival isolates was 100%. In contrast, concordance for revaccination isolates was 72.7%, with very major errors identified in 3.8% of classifications and minor errors in 22.7% of classifications of those same isolates. For ceftiofur, concordance for the arrival isolates was 100%. Similarly, concordance between revaccination isolates was 95.5%, and minor errors were found in 4.5% of classifications. For enrofloxacin, 100% concordance was found between the methods at both time points. For tilmicosin, concordance between methods for arrival isolates was 69.2%, and very major errors were found in 3.8% of classifications and minor errors were found in 26.9% of classifications. For tulathromycin, concordance for arrival isolates was 76.9%. All discordant results were minor errors. In contrast, concordance for revaccination isolates was 95.5% and again all discordant results were minor errors.

**TABLE 5:** Concordance between disk diffusion and broth microdilution of 48 *Mannheimia haemolytica* isolates

		Disk diffusion/broth microdilution susceptibility (*S*, *I*, or *R*)										No. isolates (%)		Error type^a^		
Antimicrobial	Timepoint	*S*/*S*	*S*/*I*	*S*/*R*	*I*/*S*	*I*/*I*	*I*/*R*	*R*/*S*	*R*/*I*	*R*/*R*	*P*‐value	Concordant	Discordant	Very major	Major	Minor
FLOR	Arrival	26	0	0	0	0	0	0	0	0	1	26 (100%)	0	0	0	0
	Revacc	7	5	1	0	1	0	0	0	8	.06	16 (72.7%)	6 (27.3%)	1 (3.8%)	0	5 (22.7%)
CEF	Arrival	26	0	0	0	0	0	0	0	0	1	26 (100%)	0	0	0	0
	Revacc	21	0	0	1	0	0	0	0	0	1	21 (95.5%)	1 (4.5%)	0	0	1 (4.5%)
ENR	Arrival	26	0	0	0	0	0	0	0	0	1	26 (100%)	0	0	0	0
	Revacc	0	0	0	0	0	0	0	0	22	1	22 (100%)	0	0	0	0
TIL	Arrival	17	2	1	5	1	0	0	0	0	.5	18 (69.2%)	8 (30.8%)	1 (3.8%)	0	7 (26.9%)
	Revacc	0	0	0	0	0	0	0	0	22	1	22 (100%)	0	0	0	0
TUL	Arrival	20	0	0	6	0	0	0	0	0	.06	20 (76.9%)	6 (23.1%)	0	0	6 (23.1%)
	Revacc	0	0	0	0	0	0	0	1	21	1	21 (95.5%)	1 (4.5%)	0	0	1 (4.5%)

*Notes:* Concordance for arrival isolates assessed out of 26 isolates, concordance for revaccination isolates assessed out of 22 isolates. Susceptibility interpretation indicated as susceptible (*S*), intermediate (*I*), and resistant (*R*).

Abbreviations: CEF, ceftiofur; ENR, enrofloxacin; FLOR, florfenicol; TIL, tilmicosin; TUL, tulathromycin.

^a^ Very major = classified as susceptible by disk diffusion but as resistant by broth microdilution; Major = classified as resistant by disk diffusion but as susceptible by broth microdilution; Minor = classified as intermediate by only one method.

### 3.2 Disk diffusion vs WGS

Concordance between disc diffusion and WGS is presented in Table 6. For florfenicol, concordance for arrival isolates was 100% and concordance for revaccination isolates was 95.5%. A minor error was seen with 1 revaccination isolate. For ceftiofur, enrofloxacin, and gamithromycin, concordance was 100% for both arrival and revaccination isolates. For tilmicosin, concordance for arrival isolates was 73.1% and for revaccination isolates was 100%. With arrival isolates, all discordant classifications were minor errors (26.9%). A significant difference was found in the classification of susceptibility of arrival isolates to tilmicosin between disk diffusion and WGS (*P* = .03). For tulathromycin, concordance for arrival isolates was 80.8% and for revaccination isolates was 100%; all discordant classifications in the arrival isolates were minor errors (19.2%).

**TABLE 6:** Concordance between disk diffusion and WGS of 48 *Mannheimia haemolytica* isolates

	Genotype (+ or −)/disk diffusion susceptibility (*S*, *I*, or *R*)^b^								No. isolates (%)		Error type^a^		
Antimicrobial	Timepoint	−/*S*	+/*S*	−/*I*	+/*I*	−/*R*	+/*R*	*P*‐value	Concordant	Discordant	Very major	Major	Minor
FLOR	Arrival	26	0	0	0	0	0	1	26 (100%)	0	0	0	0
	Revacc	13	0	1	0	0	8	1	21 (95.5%)	1 (4.5%)	0	0	1 (4.5%)
CEF	Arrival	26	0	0	0	0	0	1	26 (100%)	0	0	0	0
	Revacc	22	0	0	0	0	0	1	22 (100%)	0	0	0	0
ENR	Arrival	26	0	0	0	0	0	1	26 (100%)	0	0	0	0
	Revacc	0	0	0	0	0	22	1	22 (100%)	0	0	0	0
TIL	Arrival	19	0	7	0	0	0	.03	19 (73.1%)	7 (26.9%)	0	0	7 (26.9%)
	Revacc	0	0	0	0	0	22	1	22 (100%)	0	0	0	0
GAM	Arrival	26	0	0	0	0	0	1	26 (100%)	0	0	0	0
	Revacc	0	0	0	0	0	22	1	22 (100%)	0	0	0	0
TUL	Arrival	21	0	5	0	0	0	.06	21 (80.8%)	5 (19.2%)	0	0	5 (19.2%)
	Revacc	0	0	0	0	0	22	1	22 (100%)	0	0	0	0

*Notes:* Concordance for arrival isolates assessed out of 26 isolates, concordance for revaccination isolates assessed out of 22 isolates. Susceptibility interpretation indicated as susceptible (*S*), intermediate (*I*), and resistant (*R*).

Abbreviations: CEF, ceftiofur; ENR, enrofloxacin; FLOR, florfenicol; GAM, gamithromycin; TIL, tilmicosin; TUL, tulathromycin.

^a^ Very major = negative for resistance gene but classified as resistant by disk diffusion; Major = positive for resistance gene but classified as susceptible by disk diffusion; Minor = classified as intermediate by disk diffusion.

^b^ Phenotype (*S* or *R* or *I*)/genotype (−) = Any susceptibility phenotype and negative for all macrolide resistance genes or phenotype (*S* or *R* or *I*)/genotype (+) = Any susceptibility phenotype and positive for any one macrolide resistance gene.

### 3.3 Broth microdilution vs WGS

Concordance between broth microdilution and WGS is presented in Table 7. For ceftiofur, oxytetracycline, enrofloxacin, and danofloxacin, concordance between broth microdilution and WGS was 100%. For florfenicol, concordance between the 2 methods for the arrival isolates was 100%. In contrast, concordance for revaccination isolates was 68.2%, with 4.5% very major errors and 27.3% minor errors. For penicillin, concordance between broth microdilution and WGS in the arrival isolates was 42.3%; of the discordant results, 7.7% were very major errors and 50% were minor errors. Concordance for the revaccination isolates was 86.4%, with 13.6% minor errors. For tilmicosin, concordance between the testing methods in the arrival isolates was 84.6%. Of the discordant results for these arrival isolates, 3.8% were very major errors and the 11.5% were minor errors. In contrast, concordance for the revaccination isolates was 100%. For tulathromycin, concordance between the testing methods in the arrival isolates was 100%. In the revaccination isolates, concordance was 95.5%; all discordant results were minor errors (4.5%).

**TABLE 7:** Concordance between broth microdilution susceptibility and WGS of 48 *Mannheimia haemolytica* isolates

		Genotype (+ or −)/broth microdilution susceptibility (*S*, *I*, or *R*)^b^							No. isolates (%)		Error type^a^		
Antimicrobial	Occasion	−/*S*	+/*S*	−/*I*	+/*I*	−/*R*	+/*R*	*P*‐value	Concordant	Discordant	Very major	Major	Minor
FLOR	Arrival	26	0	0	0	0	0	1	26 (100%)	0	0	0	0
	Revacc	7	0	6	0	1	8	.03	15 (68.2%)	7 (31.8%)	1 (4.5%)	0	6 (27.3%)
CEF	Arrival	26	0	0	0	0	0	1	26 (100%)	0	0	0	0
	Revacc	22	0	0	0	0	0	1	22 (100%)	0	0	0	0
PEN	Arrival	11	0	13	0	2	0	0	11 (42.3%)	15 (57.7%)	2 (7.7%)	0	13 (50%)
	Revacc	0	0	0	3	0	19	.2	19 (86.4%)	3 (13.6%)	0	0	3 (13.6%)
OXY	Arrival	25	0	0	0	0	1	1	26 (100%)	0	0	0	0
	Revacc	0	0	0	0	0	22	1	22 (100%)	0	0	0	0
ENR	Arrival	26	0	0	0	0	0	1	26 (100%)	0	0	0	0
	Revacc	0	0	0	0	0	22	1	22 (100%)	0	0	0	0
DANO	Arrival	26	0	0	0	0	0	1	26 (100%)	0	0	0	0
	Revacc	0	0	0	0	0	22	1	22 (100%)	0	0	0	0
TIL	Arrival	22	0	3	0	1	0	.5	22 (84.6%)	4 (15.4%)	1 (3.8%)	0	3 (11.5%)
	Revacc	0	0	0	0	0	22	1	22 (100%)	0	0	0	0
TUL	Arrival	26	0	0	0	0	0	1	26 (100%)	0	0	0	0
	Revacc	0	0	0	1	0	21	1	21 (95.5%)	1 (4.5%)	0	0	1 (4.5%)

*Notes:* Concordance for arrival isolates assessed out of 26 isolates, concordance for revaccination isolates assessed out of 22 isolates. Susceptibility interpretation indicated as susceptible (*S*), intermediate (*I*), and resistant (*R*).

Abbreviations: CEF, ceftiofur; ENR, enrofloxacin; FLOR, florfenicol; GAM, gamithromycin; TIL, tilmicosin; TUL, tulathromycin.

^a^ Very major = negative for resistance gene but classified as resistant by broth microdilution; Major = positive for resistance gene but classified as susceptible by broth microdilution; Minor = classified as intermediate by broth microdilution.

^b^ Phenotype (*S* or *R* or *I*)/genotype (−) = Any susceptibility phenotype *and* negative for all macrolide resistance genes or phenotype (*S* or *R* or *I*)/genotype (+) = Any susceptibility phenotype *and* positive for any one macrolide resistance gene.

## 4 DISCUSSION

The availability of reliable and reproducible AST methods is essential for development of effective treatment protocols and antimicrobial stewardship programs. This is especially important in cattle with BRD, because the increase in prevalence of extensively drug resistant (XDR) strains of *Mh* has complicated disease control and treatment strategies.^2^, ^3^, ^29^ Broth microdilution has been considered the reference method for AST because it provides a numerical assessment of in vitro antimicrobial activity, and summary statistics can provide quantitative data that can be used in development of treatment protocols and also in surveillance or epidemiologic monitoring programs.^10^ We chose disk diffusion and WGS for comparison to broth microdilution for several reasons. First, disk diffusion is commonly employed by diagnostic laboratories, and surveys have shown that it is the AST method used most often in these settings.^5^, ^6^, ^7^ Second, WGS has the potential to provide additional data beyond antimicrobial susceptibility, and can enhance our understanding of microbial pathogenesis and pathogen transmission.^8^ As a result, more data are needed regarding the relative concordance between these methods so that interpretation can be made appropriately in clinical scenarios.

In our study, concordance among the different AST methods varied substantially in some cases. In addition, the variation in concordance among methods was dependent upon the antimicrobial evaluated and sampling time. More specifically, concordance was lower for tilmicosin, tulathromycin, and florfenicol among the different testing methods than was seen with other antimicrobial classes, and much of the discordance seen was specific to a given sampling time point. For example, concordance for tilmicosin and tulathromycin was lower in arrival isolates across all testing methods, whereas concordance for florfenicol was lower in revaccination isolates. In addition, concordance for penicillin between WGS and broth microdilution was only 42% for arrival isolates but 86% for isolates collected at revaccination. Although most of the discordant results were minor errors, this difference in interpretation still could alter a clinician's choice of antimicrobial and potentially impact treatment outcome adversely. Consequently, our results suggest that bacterial strain and carriage of specific resistance determinants, along with history of previous antimicrobial exposure, might impact the utility and interpretation of AST results for certain classes of antimicrobials.

Generally speaking, concordance between disk diffusion and broth microdilution was not as high as expected, and for some testing methods, fell below the minimum level of concordance considered acceptable by the United States Food and Drug Administration (FDA) for the number of isolates tested.^30^ Although only 2 isolates were classified as discordant with very major errors, numerous minor errors occurred in which an isolate was classified as intermediate by 1 of the methods evaluated. Similar work comparing agreement between disk diffusion and agar dilution for classifying in vitro susceptibility of *Mh* found that agreement between methods for florfenicol, ceftiofur, and enrofloxacin susceptibility was 100%, whereas agreement for tetracycline was only 85.3%.^31^ Although not the same methodology, agar dilution uses the same breakpoint values for *Mh* susceptibility categorization as does broth microdilution. Similar discrepancies to those reported here have been noted when comparing susceptibilities determined by broth dilution methods to disk diffusion with other organisms, particularly *Rhodococcus equi*.^32^ Other work evaluating agreement between broth microdilution and disk diffusion for assessing in vitro susceptibility of *Mh* using a limited number of antimicrobial agents has found that broth microdilution is far superior for detecting resistance, whereas either test is sufficient for classifying isolates as susceptible, although previous work by the same author found the opposite to be true.^33^, ^34^ The aforementioned studies, however, tested a limited number of antimicrobials and based their conclusions on interpretive criteria that were not always validated for respiratory pathogens of cattle, and these factors might limit application to other populations. Although the reasons for the discrepancies seen in our study are not clear, much of the discordance was seen in isolates collected at specific sampling points. Specific strains of *Mh* harbor integrative conjugative elements (ICEs) that can predominate after antimicrobial exposure.^4^, ^9^ Thus, carriage of ICEs might influence bacterial growth patterns and impact interpretation. Another reason for these discrepancies is that care must be taken when evaluating the results of disk diffusion, particularly if zones of inhibition are hazy or if small colonies are present within the larger zone of inhibition, because these factors might hinder accurate interpretation by automated plate readers.^10^ Furthermore, visual inspection after the review of equivocal results from automated readers might affect zone measurement. In these cases, a seemingly negligible difference in zone diameter could alter interpretation of results for a specific drug.

When considering WGS and its performance relative to other AST methods, fewer errors of any type were seen with disk diffusion compared to WGS than with broth microdilution compared to WGS. More specifically, very major errors were seen with florfenicol, penicillin, and tilmicosin when comparing WGS to broth microdilution. A high level of very major errors is concerning, because by definition, isolates are being classified as falsely susceptible relative to the reference method and could lead to the use of an ineffective drug. These same errors were not detected when comparing WGS to disk diffusion. Although the results of this comparison might seem promising for the future use of WGS, disk diffusion is an indirect, qualitative method that must be calibrated against broth dilution standards. Furthermore, the validated interpretations available for broth dilution are not always available for disk diffusion testing. As a result, more data are needed before gauging the potential of WGS for assessing susceptibility and resistance in organisms such as *Mh*.

Similar to our findings, earlier studies have reported a wide range of concordance for broth microdilution and WGS for AST of *Mh*. In 1 study, concordance for penicillin was 81%, for danofloxacin and tilmicosin concordance was 77%, and for enrofloxacin, florfenicol, oxytetracycline, and tulathromycin concordance was 100%.^18^ Interestingly, concordance for ceftiofur was found to be only 31%.^18^ An intermediate category of susceptibility, however, was not included and specific types of errors were not determined in that study.^18^ Nevertheless, this study similarly illustrates the difficulties in applying the complexities of genomic resistance to clinical scenarios and shows that the presence or absence of a specific gene is not always sufficient to predict phenotypic antimicrobial susceptibility. Moreover, the discordance seen in our study is higher than reported for some pathogens affecting humans and suggests that the concordance between WGS and other AST methods might be species‐specific.^12^, ^13^, ^14^, ^15^, ^16^, ^17^

Moving forward, several assumptions must be made if WGS is to be relied on to accurately predict a given resistance phenotype. Most importantly, we must assume that no epistasis is present.^11^ In other words, whether a single gene determines the resistance phenotype or if a resistance phenotype is controlled by many genes, these genes or separate gene loci must not interact in a complex or antagonistic manner. A good example of fulfillment of this assumption would be the genes controlling macrolide and tetracycline susceptibility.^11^ For these drug classes, known resistance genes all work together to confer phenotypic resistance and do not work against each other in an overly complicated manner. Other assumptions that must be considered are that resistance genes are highly expressed, the origin of a given strain has no impact on gene expression, and that all of the genes responsible for resistance in a microbe of interest are known.^11^

Regardless of the method evaluated, the number of errors, particularly very major errors, detected in our study is concerning and falls outside of the target accuracy established by the FDA for the number of resistant isolates tested.^30^ These errors could have consequences for animal treatment, because they may lead to selection of an antimicrobial that is ineffective and has a higher likelihood of treatment failure. In addition, depending on the method used to monitor resistance trends in an antimicrobial resistance surveillance program, misinterpretations might negatively impact both policy recommendations and resistance containment interventions. As a result, clinicians should interpret the results of AST considering the animal's treatment history and knowledge of the infecting bacterial agent. Furthermore, although the AST methods currently used by diagnostic laboratories are standardized, differences among laboratories and laboratory technicians may introduce errors into the testing process and alter the results obtained by a specific method. In addition, factors intrinsic to the microbe, such as the ability to form biofilms and to form persister cells, could make assessing phenotypic susceptibility more difficult.^11^, ^35^, ^36^ Thus, it is important to remember that CLSI interpretation guidelines may not always perfectly predict clinical outcome. Susceptibility breakpoints take in vitro test results and extrapolate them into an in vivo response. In clinically ill animals, factors such as host immune status, disease duration and severity, physiologic variations, and intermicrobial interactions can affect bacterial growth and gene expression differently than factors present in a laboratory setting.^11^, ^37^

## 5 CONCLUSION

Antimicrobial susceptibility testing is a powerful tool that allows more informed treatment decisions. Our results show that important discrepancies in the results of AST exist and depend on the method used, the bacterial strain being evaluated, and the previous history of antimicrobial exposure. More specifically, many misclassifications are dependent on the antimicrobial being tested, the time point being evaluated, and the test being used. These data suggest that harmonization of AST methods for *Mh* is needed so that clinical utility can be optimized.

## CONFLICT OF INTEREST DECLARATION

Authors declare no conflict of interest.

## OFF‐LABEL ANTIMICROBIAL DECLARATION

Authors declare no off‐label use of antimicrobials.

## INSTITUTIONAL ANIMAL CARE AND USE COMMITTEE (IACUC) OR OTHER APPROVAL DECLARATION

Authors declare no IACUC or other approval was needed.

## HUMAN ETHICS APPROVAL DECLARATION

Authors declare human ethics approval was not needed for this study.
